# Nipah Virus Disease: Epidemiological, Clinical, Diagnostic and Legislative Aspects of This Unpredictable Emerging Zoonosis

**DOI:** 10.3390/ani13010159

**Published:** 2022-12-31

**Authors:** Luigi Bruno, Maria Anna Nappo, Luca Ferrari, Rosanna Di Lecce, Chiara Guarnieri, Anna Maria Cantoni, Attilio Corradi

**Affiliations:** 1 Department of Prevention, Azienda Sanitaria Locale (A.S.L.) Napoli 3 Sud, 80053 Castellammare di Stabia, Italy; 2 Department of Veterinary Science, University of Parma, 43126 Parma, Italy

**Keywords:** Nipah virus, epidemiology, pathology, immune response, diagnosis, vaccines, legislation

## Abstract

**Simple Summary:**

This review summarizes fundamental aspects of Nipah virus (NiV) infection with particular emphasis on disease in swine and its zoonotic potential, focusing on epidemiology, clinical signs, diagnosis, prevention and legislation. The main outbreaks were reported in Southeast Asia, and, in humans and pigs, a severe disease can be characterized by respiratory and neurological signs, and high mortality. This viral infection has, like other recent infections, a natural reservoir in bats, but human-to-human transmission has been also recorded. Effective treatments and prophylaxis are still main goals due to the still scarce knowledge on the infection in humans with regards to the pathogenetic- and immunological-related features of the disease. The effects of climate change should be considered together with the anthropogenic pressure on ecosystems where zoonoses can spread. Action and surveillance plans should be continuously employed to monitor areas at risk as, as today they represent an essential preventive measure to avoid the onset and diffusion of outbreaks, even in countries that have direct/indirect relationships with countries at risk.

**Abstract:**

Nipah virus (NiV) infection is a viral disease caused by a Henipavirus, belonging to the Paramyxoviridae family, responsible for a zoonosis. The course of the disease can be very serious and lead to death. NiV natural hosts are fruit bats (also known as megabats) belonging to the Pteropodidae family, especially those of the *Pteropus* genus. Natural infection in domestic animals has been described in farming pigs, horses, domestic and feral dogs and cats. Natural NiV transmission is possible intra-species (pig-to-pig, human-to-human) and inter-species (flying bat-to-human, pig-to-human, horse-to-human). The infection can be spread by humans or animals in different ways. It is peculiar how the viral transmission modes among different hosts also change depending on the geographical area for different reasons, including different breeding methods, eating habits and the recently identified genetic traits/molecular features of main virus proteins related to virulence. Outbreaks have been described in Malaysia, Singapore, Bangladesh, India and the Philippines with, in some cases, severe respiratory and neurological disease and high mortality in both humans and pigs. Diagnosis can be made using different methods including serological, molecular, virological and immunohistochemical methods. The cornerstones for control of the disease are biosecurity (via the correct management of reservoir and intermediate/amplifying hosts) and potential vaccines which are still under development. However, the evaluation of the potential influence of climate and anthropogenic changes on the NiV reservoir bats and their habitat as well as on disease spread and inter-specific infections is of great importance. Bats, as natural reservoirs of the virus, are responsible for the viral spread and, therefore, for the outbreaks of the disease in humans and animals. Due to the worldwide distribution of bats, potential new reports and spillovers are not to be dismissed in the future.

## 1. Introduction

Nipah virus (NiV) infection is a viral disease caused by a virus belonging to the Henipavirus genus, in the Paramyxoviridae family, responsible for a zoonosis whose main clinical manifestations in humans are respiratory and neurological. It can be defined as an emerging zoonosis. Zoonotic pathogens account for 60% of emerging infectious diseases, of which about 70% originate from wildlife [1]. The novel RNA paramyxovirus (genus Henipavirus), closely related to the Hendra virus, was named after the outbreak in the village of Sungai Nipah in the State of Negeri Sembilan (close to the Federal Territory of Kuala Lumpur), Malaysia, in which the virus was first isolated from a human patient in 1998 [2]. The course of the disease can be very serious and lead to death. The disease is also widely described in pigs, with clinical signs involving the respiratory and nervous systems [3]. The natural reservoir of the virus is represented by frugivorous bats called “flying foxes” belonging to the *Pteropus* genus. The main outbreaks of NiV infection have so far occurred in the geographical area of the carrier bats, i.e., in Malaysia, Singapore, Bangladesh and India, but cases were also described in the Philippines. The emergence of the virus and the zoonotic potential of transmission to animals and humans seem to be related to losses in the bats’ habitat [4]. The modes of transmission are different: *Pteropus*-swine-man, human contagion through the consumption of contaminated food, and inter-human contagion; direct bat-human contagion is also hypothesized. When outbreaks occur in pigs, the only possible measures are isolation, blocking of movements and killing of infected animals [5,6]. A definitive diagnosis can be performed by using molecular techniques, but serological tests are also available. NiV infection is an emerging and potentially dangerous disease whose spread must be curbed; currently, the main strategy is prevention via limiting contacts with reservoir species, using proper pig farming practices and improving the hygiene and feeding habits of some populations living in areas where the disease occurs [5]. Nowadays, drugs and vaccines and/or vaccine candidates in various species are scarcely employed or under investigation, therefore further studies are necessary to improve the knowledge about the interaction between NiV and the immune response, apply prevention methods, and develop effective prophylactic and curative therapies. In particular, due to the high risk in manipulating the virus itself in challenge animal studies, several experimental vaccines based on the main NiV immunogenic proteins or peptides have been developed mainly to investigate their immunogenicity, that is their ability to elicit a neutralizing antibody response potentially able to rapidly counteract the infection and induce a cell-mediated response to mediate viral clearance [7,8,9]. However, some experimental vaccines also elicited a response to varying degrees of clinical protection in model animals [3,10,11,12,13].

Furthermore, a better understanding of the spatial habitat preferences and diffusion of reservoir bats would be a useful contribution to their conservation and would help better prevent potential disease transmission [4]. Remote sensing (RS) and geographic information system (GIS) technologies have proven to be useful tools in investigating animal distribution in relation to environment/human settlements and diseases in wildlife [14,15], including the characteristics of the flying fox colonies potentially carrying NiV and their neighborhoods [4,16].

However, climate change, bringing an extreme increase in air temperature in tropical monsoon areas, has an adverse effect on the adaptive capacity of flying foxes to new environmental conditions, especially in relation to demography and species survival and thus NiV diffusion [17,18,19].

In Europe, the relative legislation refers to the generic management of communicable diseases, and specifically to the articles listed in the Regulation 2016/429 [20] about emerging diseases. In Malaysia, following the NiV outbreaks in pigs, a series of provisions aimed at containing the spread of the infection were planned, such as the strict isolation of the potential disease transmitters, the prohibition of the pig and meat trade, the establishment of an infected area and a protection area around the outbreaks, and the killing of infected pigs [2,21]. In India, following the recent cases in humans, the Indian National Centre for Disease Control (NCDC) established guidelines for the definition of suspected, probable and confirmed cases of NiV infection in order to detect and control the outbreaks early and subsequently regulate several public health activities aimed at containing the spread of the infection [3,22].

Therefore, a “One Health” approach, considering humans, domestic and peridomestic animals as well as the environment, is required to effectively control the disease.

## 2. Biological Features of the Nipah Virus (NiV)

NiV is a virus belonging to the Henipavirus genus, in the Paramyxoviridae family and Orthoparamyxovirinae subfamily [23,24]. The only other member of the genus endowed with pathogenicity and zoonotic potential is the Hendravirus (HeV) [25,26,27] identified in the 1990s following the death of several people after contact with infected equines in Australia [28]. At present, reports of spillover phenomena of the Henipavirus disease in humans are limited to Southeast Asia, although the widening of surveillance has allowed to identify these viruses in chiroptera sera in sub-Saharan Africa and Brazil [29,30,31,32,33,34]. Furthermore, in a recent study on sera from humans and bats in Cameroon, about 3–4% of human samples were found to be seropositive. Particularly, they were individuals who were involved in the butchering of bat meat [35]. Antibodies produced against NiV also cross-react with the HeV but not with other members of this family [36]. One of the most important biological characteristics of henipaviruses is the broad host spectrum both in vivo and in vitro [37]. Bats can be the main reservoir for some henipaviruses, which can cause a spontaneous disease in humans, pigs, horses, dogs and cats. 

**Figure 1:**
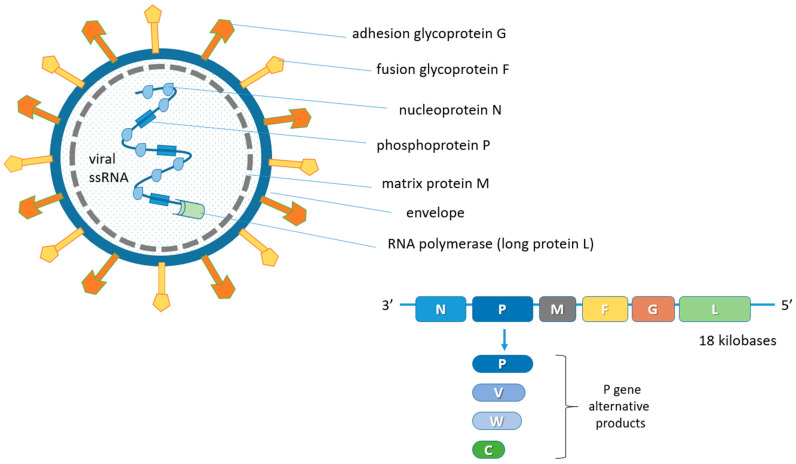
Schematic representation of the NiV structure and viral genome organization. N, P and L proteins interact with the unsegmented single-stranded negative-sense viral RNA genome forming the ribonucleoprotein (RNP) complex. The matrix M protein, used for viral assembly and budding, is associated with the inner side of the envelope which contains the G and F glycoproteins for adhesion to ephrin-B2 and -B3 receptors and fusion, respectively. The P gene products (V, W and C proteins) derive from mRNA editing and alternative open reading frames (ORF). The six coded genes are flanked by a 3′ leader and a 5′ trailer region.

Paramyxoviruses are enveloped pleomorphic viruses with an unsegmented single-stranded negative-sense RNA genome, encoding six structural and three accessory proteins [24,38,39]. These include the genome codes for nucleoprotein (N), phosphoprotein (P), matrix protein (M), fusion protein (F), adhesion glycoprotein (G) and for large protein (L) or RNA polymerase in the order 3′-N-P-M-F-G-L-5′ [40,41,42]. The genome of some viruses of the Paramyxoviridae family codes for cell attachment proteins which can exert hemagglutination (H) and neuraminidase (N) functions. Specifically, the NiV G protein is involved in the adsorption phase but does not display H functions. In addition, the P gene also provides the three non-structural proteins V, W and C by means of mRNA editing mechanisms and alternative open reading frames (ORF) (Figure 1) [5,43]. 

Non-structural proteins play a crucial role in virulence by indirectly inhibiting the action of immune interferons [44,45]. The virus is classified as a BSL-4 (biosafety level-4) pathogen, due to its high pathogenicity and the absence of effective treatments or vaccines [46,47,48].

The exact length of the NiV genomic sequence is described in multiple isolations [44,45,49]. The complete viral genome consists of approximately 18 kilobases (kb) [39,50] and, like the HeV genome, is significantly longer than that of other members of Paramyxoviridae (about 155 kb on average) [51].

The P gene of the HeV and NiV contains an mRNA editing site that is identical to that of the measles virus [44]. A single G insertion allows the expression of the V protein, while two G insertions allow the expression of a protein comparable with the Sendai virus W protein [44,52,53,54].

Both the NiV and HeV do not show hemagglutinin and neuraminidase activities, which are a common feature of many paramyxoviruses. Furthermore, they share a broad host spectrum unlike that of the other viruses of the family they belong to [43,55].

Several variants have been observed in human NiVs sampled from various outbreaks in Malaysia, India and Bangladesh. Similarly, the NiVs isolated from chiroptera samples collected in different geographic areas revealed different genomic variations [45,56,57].

The interaction of paramyxoviruses with target cells is mediated via adsorption using the G, H or HN proteins and the transmembrane fusion F protein. Specifically, the NiV uses glycoprotein G, which exploits the ephrin-B2 or, to a lesser extent, ephrin-B3 receptors [58,59,60]. The Ephrin-B2 receptors are mainly expressed in endothelium, smooth muscle and the brain, followed by the lungs, placenta and prostate [61]. The distribution of these receptors explains the clinical-pathological characteristics observed during the clinical signs of the disease.

The ephrin-B2 and -B3 receptors play a fundamental role in the migration of neuron precursors during embryogenesis [62] and for this reason they are highly conserved in the different classes of animals, sharing about 96% homology with proteins [63].

The NiV can survive up to 3 days in some fruit juices or mangoes and at least 7 days in date sap kept at 22 °C. The virus resists for approximately 18 h in the urine of reservoir bats. The pathogen is relatively stable in the environment and remains viable at 70 °C for 1 h and is completely inactivated by heat at 100 °C for longer than 15 min [46,47]. The NiV is inactivated by common disinfectants such as sodium hypochlorite [64].

## 3. Host Range

**Figure 2:**
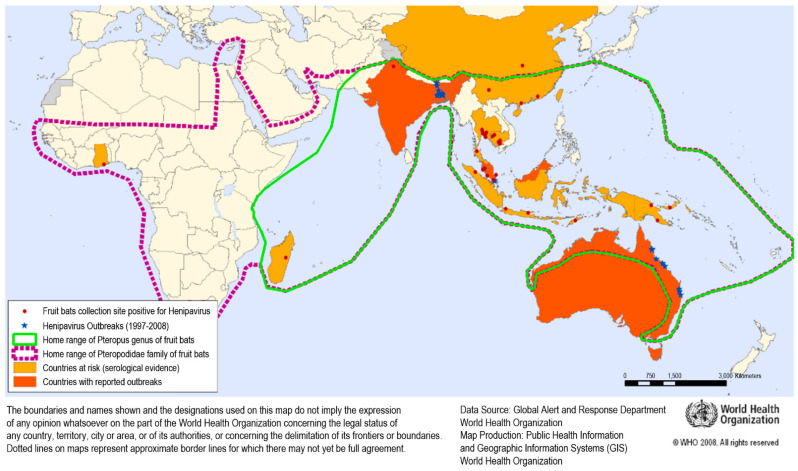
Geographic distribution of Henipavirus outbreaks and fruit bats of the Pteropodidae family. Countries at risk of outbreaks based on collection of positive serological specimens from fruit bats (orange) and countries with reported outbreaks (red) are shown. Violet spots indicate collection sites of fruit bats that resulted positive for Henipavirus, while blue stars indicate sites where Henipavirus outbreaks occurred between 1997 and 2008. Areas indicating the home range of the *Pteropus* genus of fruit bats (delimited by the green solid contour) and the home range of the Pteropodidae family of fruit bats (areas delimited by the violet dotted contour) are shown. Data source: Global Alert and Response Department, World Health Organization, WHO; map production: Public Health Information and Geographic Information Systems (GIS). Reproduced and adapted according to WHO permission ID: 390902 [65].

Natural hosts of the NiV are fruit bats (also known as megabats) belonging to the Pteropodidae family, especially those of the *Pteropus* genus. Flying foxes are the fruit bats mostly involved in NiV spread, with direct shedding to receptive animals, human beings included, or through NiV-contaminated palm fruits (or palm sap) or biological bat matrixes (e.g., urine, faeces). Megabats represent NiV reservoirs in endemic geographical areas of Southeast Asia and sub-Saharan Africa (Figure 2).

Natural infection in domestic animals has been described in farming pigs, horses, and domestic and feral cats. Natural NiV transmission can be intra-specific (pig-to-pig, human-to-human) and inter-specific (flying bat-to-human; pig-to-human and horse-to-human). Ruminants are spillover hosts in which NiV infection in the ovi-caprine is ascertained, while bovine is a species considered as NiV-permissive. Dogs are also susceptible to NiV infection, but dogs and cats do not seem to play a zoonotic role [66]. A study on NiV infection in peridomestic and feral cats in the Tioman island (Malaysia) pointed out that natural NiV infection is rare in cats and the zoonotic risk is classified as low [67]. 

To date, no data are available on the susceptibility and infection of wildlife, except for the different members of the Pteropodidae bat family. It is reported by the members of the World Organization for Animal Health (WOAH), through the voluntary annual report, that NiV infection is a non-WOAH-listed disease in wildlife [68,69].

During a Malaysian NiV outbreak, indirect evidence of non-susceptibility to NiV infection was observed in rodents and birds living in the outbreak area, proven using NiV-seronegative blood immunoglobulin testing [70].

In experimental infection animal models, a permissive NiV transmission was recognized in ferrets, guinea pigs, golden hamsters and mice, as well as in non-human primates (e.g., squirrel monkeys and African green monkeys) [71].

NiV zoonotic infection, flying fox-to-human, is possible via the inhalation of aerosol NiV virions or ingestion of NiV-contaminated palm fruits or palm sap. NiV-contaminated palm fruits are also a cause of infection in pigs and horses fed with or upon accidental food ingestion of contaminated fruit or palm sap. NiV zoonotic transmission, pig-to-human, is well recognized in pig farms and pig slaughterhouse workers, respectively during piglet and pork handling. A zoonotic NiV outbreak in the Philippines was associated with handling horse meat or horse NiV-contaminated biological fluids at slaughterhouses. Human consumers were infected by ingestion of undercooked horse NiV-contaminated meat, but also human-to-human transmission was identified [3,72].

## 4. Epidemiology

The disease can be contracted by humans or animals in different ways. It is peculiar how the pathways of contagion in the different hosts also change depending on the geographical area for different reasons including the different breeding methods and some eating habits.

In Malaysia, humans contract the disease from infected suids, which act as the intermediate host of the virus [73]. In this area, the main contagion risk factor is certainly identified in the biological promiscuity characterized by the close contact of humans with pigs and their excrements [74,75]. In Malaysia, a significant number of animals are raised in pig farms, an area in which NiV infection can spread as an inter-animal infection, but slaughterhouses also play a role in the spread/contagion, as they are places where a leap between animal species, i.e., pig-to-man, can occur. Swine is considered a biological reservoir for infection and becomes infected by consuming fruit bitten by NiV-carrying bats. During the 1998 outbreak, the dogs present in the pig farms were also found infected and this obviously represents a further source of potential risk for the transmission of NiV to humans [75]. Other factors that can facilitate the transmission of the virus are certainly linked to deforestation and climate change.

In Bangladesh, many NiV outbreaks occurred during winter, especially in the central and north-western regions of the country [76]. In these geographical areas, *Pteropus* has been identified as the NiV reservoir animal [77]. Although close contact with pigs has been hypothesized in most human patients in Bangladesh, this condition has only been ascertained in the onset of a single NiV outbreak, despite the high seroprevalence for NiV in suids tested in this country [78]. In Bangladesh, the main route of transmission of infection is related to the consumption of the raw sap of the date palm, and this feeding behavior provides scientific knowledge about the route of transmission from bat to humans [79]. Indeed, NiV outbreaks in humans often coincide with the date harvest season (from December to May). *Pteropus* bats, in fact, often come in contact with date palms and lick the spontaneous sap flows of the plant (contaminating them with NiV) which are then collected and used for food consumption by humans. Additionally, bats can contaminate collection containers with their NiV-positive biological excretions (urine and feces) [80]. NiV can survive in date palm sap and urine for days and usually the drink is consumed in a short time after collection, with a high probability of contagion [79]. The difference in Bangladesh, compared to Malaysia, is characterized by the ways of raising livestock: in Bangladesh, in fact, people manage animals in small groups of livestock, hence a lower probability of animal-to-human contagion. Seroprevalence studies on farm animals, cattle and goats, have highlighted the presence of circulating antibodies in the blood and therefore have ascertained their susceptibility to NiV infection [23,78]. Human-to-human transmission is another important route of transmission in Bangladesh and has been identified in all outbreaks: one of the main ones occurred in Faridpur in 2004 [81]. NiV is transmitted through droplets [82] and its RNA was found in the saliva of patients [44]. In hospitals, healthcare professionals and patients were at considerable risk of spreading the pathogen through coughing and sneezing in poorly ventilated areas and where the risk due to the of contact with bodily fluids or shared beds was high. In addition, crowding, the uncontrolled flow of visitors and poor hygiene practices such as scarce hand washing are other important factors that contribute to the transmission and spread of the disease [83]. Another possible source of human contagion is related to human–bat cohabitation in common geographical areas where bats can contaminate the environment with their urine. This epidemiological hypothesis is still poorly supported by scientific evidence [84,85]. The same can be said of the hypothesis regarding the direct human consumption of fruit bitten by bats. Other reported outbreaks in Bangladesh refer to the year 2012, with 12 cases and a mortality rate of 83% [86]. In Malaysia, in 8% of documented cases, infection proceeded asymptomatically [87].

Clinically, there are interesting differences between the outbreaks in Malaysia and those in India. A high mortality rate was observed in India and Bangladesh (about 70%) compared to Malaysia (where 40% of deaths occurred). The respiratory disease was observed in 70% of Indian and Bengali patients [57,88], unlike in Malaysia where there was no significant presence of such clinical signs [89]. The three viral strains that were the cause of NiV infection in the aforementioned countries are genetically different: NiV-B isolates in Bangladesh and NiV-I isolates in India belong to the B genotype, while NiV M isolates in Malaysia belong to the M genotype [44,50].

In India, in 2001 and 2007, in West Bengal, two outbreaks of the NiV in humans were recorded, both characterized by very high mortality: the first with 45 deaths out of 66 infected (68.18%) in the Siliguri district; the second with five deaths out of five infected (100%) in the Nadia district. It should be noted that in these geographical areas, the resident population does not commonly consume date sap for nutrition purposes. In 2001, in Siliguri, patient zero, although never identified, was certainly admitted to the district hospital, thus infecting 11 patients in the hospital. These patients were then transferred to other hospitals generating a further spread of the virus involving 25 healthcare workers and eight visitors [57]. All outbreaks described so far in India have shown an infectious capacity characterized by human-to-human transmission. An interesting aspect that emerged from the molecular-genetic investigations is that the viral strain of NiV circulating in Bangladesh and India differs from the Malaysian one by about six nucleotides [44,90]. The scientific interpretation of the biological-molecular difference is probably related to a separate co-evolution of the viral strains within their natural reservoir [49]. The most recent reports in the Indian subcontinent are those in 2018 in the Perambra Block, in 2019 in the Vadakkekara village, both located in the Kerala region, and in Kerala in 2021 (Table 1) [6,91,92].

An outbreak with a mortality rate of 82% was reported in the Philippines in 2014. Of particular interest is the fact that 10 patients had a history of direct contact with horses and horse meat consumption. In addition, the deaths of 10 equines were reported during the same period, nine of which exhibited neurological symptoms. Unfortunately, the deceased animals were not subjected to molecular biology tests to ascertain the presence of the NiV genome. Furthermore, unlike with the Hendra virus, at present there are no relevant reports to support the contagion of equidae with this pathogen. Five human patients, including two healthcare workers, contracted the disease through direct contact with infected people. This viral strain was genetically correlated with the Malaysian strain for which human-to-human transmission has been described as a minor source of contagion [72,93] (Table 1).

This suggests the possibility of a coevolution of different variants of the NiV in bats and the consequent increase in the likelihood of mutations and spillover events [3].

Other possible risk factors for transmission are represented by funeral rituals. During purification rituals, NiV exposure occurs after close contact with the deceased’s face and hands as the virus remains in *post mortem* respiratory secretions. During these rituals it is not culturally accepted to wear gloves or masks, increasing the likelihood of infection, especially when the person touches the face, eyes and nose during the function. In a very similar way, some religions provide for ritual baths with water to be poured directly on the body of the deceased. Contaminated water is an important source of contagion when it comes in contact with religious clothing and bodies. The proper disposal of bodies and management of contaminated bodies is key to the prevention of the pathogen spread. Furthermore, in these contexts, it would be appropriate to sensitize the population to implement measures that can somehow reduce the probability of contagion while respecting the nature of the religious rite without compromising it [94].

Human-to-human transmission is a potential public health problem and a significant route for infection. This can happen through direct contact with infected individuals or their secretions. Direct contact can occur from physical contact or interaction with the patient for necessary medical-nursing procedures [95]. Respiratory secretions and saliva carry the greatest risk [96]. Another major source of contagion is sharing dishes and drinking from the same glass with a sick person, which can facilitate transmission through saliva. Overcrowded bedrooms due to the lack of space and poverty or the willingness of families and friends to be in close contact with the infected person can expose other family and community members to the virus, leading to a chain of transmission. Isolation is the key measure in these situations, particularly to avoid the exposure to respiratory and urinary secretions [97].

Except for the date palm sap and human-to-human transmission, other hypotheses regarding the modes of transmission are possible. A mode may be bat hunting and consumption of bushmeat, reported during the outbreaks in Bangladesh [98]. In this study, around 49% of villages reported bat hunting for either consumption or medicinal uses.

NiV-affected village members reported bats eating fruit from trees such as mango, guava and litchi. Whether this contaminated fruit served as vehicles of transmission is not known but deserves further study as theoretically it seems to be a plausible route of transmission. In areas such as Kerala, coconut palm is used to make ‘toddy,’ which is a popular alcoholic beverage [99]. It is suspected that coconut palm ‘toddy’ is also a means of NiV transmission. 

In addition, recreational activities related to tourism in countries at risk, in which people are suspected of being in contact with (potentially infected) frugivorous bats (e.g., photographs), might be implicated in the transmission of the virus by self-contamination due to the contact between contaminated hands and the secretions or mucosae of the mouth, nose and eyes (Ferrari L., personal observations, Indonesia, July–August 2022).

It was demonstrated that infectivity is higher with individuals that have respiratory symptoms [66]. An additional way of human-to-human transmission, needing further investigation, is the sexual route. The virus was found in the semen of infected individuals [100] using polymerase chain reaction (PCR) at 2 weeks after clearance from blood and urine in a survivor of an outbreak in Kerala. As virus isolation was not carried out in that case, the virus infectivity could not be ascertained. Also, the variation of the strains may have a role in transmission. The Bangladesh strain reached higher levels in the blood and saw increased shedding in the oral and respiratory secretions in an animal model as compared to the Malaysian strain. This could possibly explain the higher secondary attack rates in the Indo-Bangladesh outbreaks with higher mortality [96].

## 5. Biological Reservoir

The NiV has chiroptera belonging to the genus *Pteropus* (Erxleben, 1777) as its natural biological reservoir [2,79,101,102,103]. It has also been reported in the straw-colored fruit bat *Eidolon helvum* that is widespread in sub-Saharan Africa [104].

**Figure 3:**
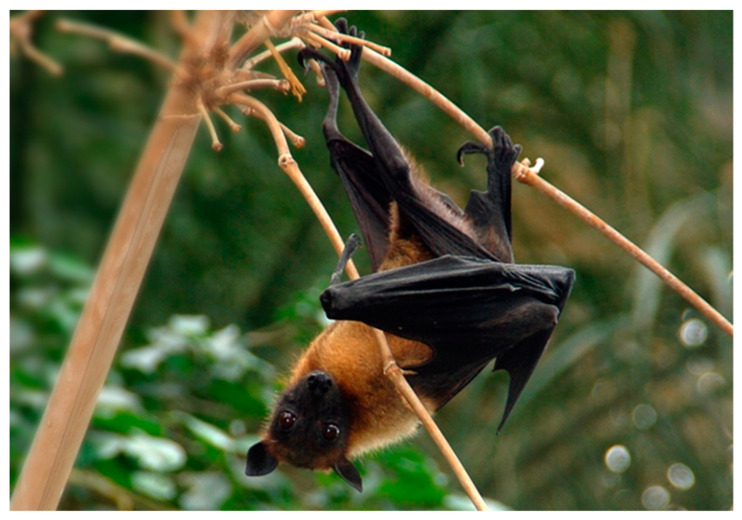
*Pteropus giganteus*, main reservoir of NiV (Fritz Geller-Grimm, 2002, Zoological Garden Berlin, Germany). This photograph is licensed under the Creative Commons Attribution-Share Alike 2.5 Generic license [105].

The genus *Pteropus* includes the largest bat species on the planet, which have the following morphological characteristics: The length of the forearm ranges from 86 mm for *P. personatus* to 220 mm for *P. vampyrus*. The latter species can have a wingspan of about 1.8 m. Some of these species can reach a weight of 1.6 kg. The fur can be extremely short and sparse or long and thick. The overall body color can range from silvery white to black. There is always a part of the fur on the shoulders, called the “mantle”, of a different color from the back, and generally brighter. Some species are characterized by face masks. The muzzle is long and tapered, the eyes are large. The ears can be small and partially hidden in the fur or long and pointed. The second finger always presents a claw, while the wing membranes are attached posteriorly to the second toe. The uropatagium is not fully fledged in most species. It has no tail, while the calcar is well-developed (Figure 3).

The genus is widespread in Madagascar, in several islands of the western Indian Ocean and in Asia, from the Indian subcontinent throughout Indochina, Indonesia, Malaysia, the Philippines and the Ryukyu Islands, even as far as Australia, Melanesia, Polynesia and the Cook Islands [106,107]. There are at least 60 extant species in the genus [108].

Regarding nourishment, the diet of *Pteropus* is mainly composed of fruit, flowers, nectar and leaves [109,110], but occasionally bats can ingest insects such as hemiptera belonging to the Cicadidae family [111]. In Australia, they prefer eucalyptus pollen and flowers, particularly the Melaleuca (L., 1767) and Banksia (L.f., 1782) flowers [112]. They also feed on a wide variety of crops, causing troubles to farmers. Crops eaten by flying foxes include cashew, pineapple, areca, breadfruit, jackfruit, neem, papaya, citrus, fig, mango, banana, avocado, guava, sugar cane, tamarind and grapes [113]

Cappelle et al. in 2020 [114] identified seven plant species from faecal samples of *P. lyley* collected during the study. These included pollen from flowers of the Malay apple (*Syzygium* spp.), kapok (*Ceiba pentandra*), petai (*Parkia* spp.) and cotton tree (Bombax ceiba), seeds from fig trees (Ficus spp.) and fibers and pulp of sapodilla (*Manilkara zapota*) and mango (*Mangifera indica*).

Bats of this genus are universally recognized as reservoirs of zoonotic viruses; in fact, they behave as hosts of numerous agents belonging to about 15 viral families [115]. In addition, they possess special characteristics such as long-distance flight, occupation of large geographical areas, long-life and a colonizing attitude, which facilitate spillover from bats to other animals and humans [116]. The expansion of urbanized and agricultural areas increases the possibility of contact between *Pteropus* and domestic animals/humans. The knowledge of the ethology of the species is also important since these bats have a high potential for spreading viruses through their night flight activity. Hengjan and colleagues in a study in 2017 found that about half of the specimens examined were awake and exhibited varying levels of activity during the day. The main behaviors that led to the spread of the virus were self-grooming, mating/courtship/aggression, with spikes in the early morning. Males were more active and took longer in sexual activities than females. On the other hand, no difference was observed in the time spent on negative social activities between the sexes; the positive ones, especially parental care, were instead performed mainly by females. Sexual and social behaviors facilitate the exchange of fluids between bats and, consequently, the transmission of microbial agents [117].

There is now strong evidence that *Pteropus* is the NiV reservoir in Malaysia [118]. In the Tioman Island, the NiV has been isolated from the urine of bats perched on trees [102]. In any case, attempts to isolate or amplify viral sequences in subjects captured in Malaysia near the outbreaks have not been successful [101]. Serum neutralizing antibodies have been found in 4–31% of bats species, including those from the Tioman Island. Fruit bitten by *Pteropus* and fallen into pigsties could contain enough virus to infect the pigs that feed on it, and for this reason the pig would play a key role as a NiV-amplifying host. The very close proximity of pigs in Malaysian farms contributes significantly to interspecific transmission.

*Pteropus giganteus*, the only species present in Bangladesh [119], showed antibodies against the NiV following investigations after the 2003 outbreak, demonstrating its role as a biological reservoir for the virus in the Bengal region [103]. In this region, the previous outbreaks always occurred between January and May, and were probably linked to the seasonal increase in the viral spread of NiV by bats that were attracted to the crops in that period of the year. This caused an indirect link between *Pteropus* and humans. Certainly, the peculiar eating habits in this country, in particular the consumption of raw date palm sap, another plant on which *Pteropus* lives, are implicated in the epidemiology of NiV encephalitis in Bangladesh [79].

As regards the regulatory framework of *Pteropus*, the different species are included in the Convention on International Trade in Endangered Species of Wild Fauna and Flora (CITES), Appendices I and II [120,121] regulate the trade and movements in a very restricted way. Appendix I includes species threatened with extinction that are or can be negatively influenced by the international trade. Therefore, for these species, international trade of specimens is forbidden, despite it being permitted under exceptional circumstances. Appendix II includes species not necessarily threatened with extinction, but which trade must be strictly controlled in order to avoid compromising their survival. In addition, the trade of these wild bats, such as for other wild species, is regulated by Article 3 of the EU regulation 2016/429 (the new reference point on “Animal Health” [20]) which prohibits the import, possession and trade of live animals of wild and exotic species taken from their natural environment, as well as of hybrids between these specimens and other domestic species or forms taken from their natural environment.

## 6. Climate and Anthropogenic Influence on *Pteropus* Bats and NiV Spillover Events

Habitat loss is the greatest threat to wildlife and biodiversity. The loss and fragmentation of wildlife habitats can lead to increased contact among wildlife, domestic animals and humans, potentially leading to the emergence and spread of zoonotic diseases.

Useful tools to understand spatial patterns and relationships among different variables, even apparently unrelated, are geographic information system (GIS) and remote sensing (RS) technologies. These geospatial technologies have been applied to gather information describing phenomena also in the veterinary field, such as the distribution of pathogen outbreaks and diseases in wildlife related to human behaviors and settlements as well as environmental factors and changes in climate in order to monitor and predict future pathogen spread and potential zoonotic outbreaks. These technologies help different categories of professionals and institutions to implement better management policies in a “One Health” perspective [4,14,15,16,122,123].

A better understanding of the *Pteropus* flying fox habitat preferences and diffusion would be a useful contribution to its conservation and would help better prevent potential disease transmission. In this regard, a study focused on the spatial characterization of colonies of flying foxes in Thailand using field observations, RS and GIS, showed that flying fox colonies are found particularly in locations surrounded by water, vegetation and controlled/protected religious areas. This aimed at mapping potential contact zones for bats, pigs and humans. High-risk areas for NiV zoonosis in pigs were proven to include the agricultural area around Bangkok where pig farms are numerous [4].

A recent GIS-based study on NiV-carrying bats in India and Bangladesh investigated the likely change in the distribution of *Pteropus medius* (based on data from 2015 onwards) under different future socioeconomic and environmental scenarios and the concurrent risk increase in NiV spillover events. It was predicted that the risk of NiV spillover events in India and Bangladesh will likely increase due to population growth and persistent environmental degradation. The adoption of rigorous public health measures in the areas predicted to be at major risk of NiV spillover from *Pteropus medius* to humans could reduce potential future spillovers and control future outbreaks [16].

Climate and anthropogenic changes can have important roles in the spread of viral infections to animals and humans [124]. Regarding NiV infection, it is thought that the emergence of the virus and the zoonotic transmission to susceptible animals and humans is related to losses in the reservoir bat’s habit.

Fruit bats *Pteropus* populate the climate area of tropical monsoons, category Am, of the Köppen climate classification [125]. The tropical monsoon has a characteristic yearly constant temperature of 18 °C, as a minimum climate degree, and high rainfall. Tropical monsoon climate areas favor the maintenance of the tropical forest including fruit bats’ feed reserve and bats play a role (i.e., pollinators and seed dispersers) in maintaining vital tropical forest eco-systems [19,126].

Bats manage a wide range of body thermal regulation as homeotherms or poikilotherms. Flying fox bats are able to control body temperatures adapting to environment temperatures above 30 °C by decreasing the basal metabolic rate and entering a state of “torpor”. 

The bat, a nocturnal mammal, enters a state of controlled torpor during the hottest hours of the day, reducing basal metabolic activities to a minimum to overcome heat stress. The extreme environmental thermal events were studied in Australia and a significant rate of mortality was recorded in flying fox bats when the air temperature was ≥42.0 °C [17,18,19].

However, flying fox bat geographical distribution is highly dependent on food sources, a combination of nectar, pollen and fruit, especially those produced by Eucalyptus trees in woodlands and open forests [127,128,129]; therefore, a lack of food resources due to deforestation and climate change may disperse flying foxes outside their typical habitats with consequent increased levels of stress. Studies using urine cortisol concentrations to measure the physiological stress in Pteropid bats have pointed out that lower winter temperatures increase cortisol concentrations, which are associated with HeV excretion in flying foxes [130,131]. A similar condition may occur with the NiV. Another aspect is the body condition score (BCS) that is related to the nutritional status. Poor body condition is associated with increased seroconversion and HeV infection risk in flying foxes. This increases the urinary excretion of viable virus, with an increased risk of transmission to horses, the main host of the HeV disease [132,133].

Specific weather patterns have been associated with spillover events. A number of studies have consistently demonstrated associations between dry conditions and spillover events of HeV [1,129,134,135,136,137]. El Niño cycles (warmer and drier conditions) in spring/summer correlates with spillover events in autumn/winter [129,134]. El Niño and drought events are predicted to occur more frequently in the future due to climate change [138,139]. Therefore, these extreme weather conditions coupled with the cascading effects on flying foxes may increase the risk and frequency of HeV spillover events/outbreaks and expansion into new areas. 

Field studies carried out in Malaysia have suggested a complex interaction of several factors that might have triggered the 1997–1998 outbreak: the reduction of the inhabitants of the reservoirs that are linked to deforestation for commercial and agricultural purposes, prolonged drought driven by the strong El Niño Southern Oscillation (ENSO) phenomenon, forest fires caused by man, intense promiscuity of crops and pig farms and overcrowding of pigsties. All this led to a greater probability of virus spillover from its natural reservoir to the pig and consequently to humans [102].

Regarding human behaviors and NiV infection, in a qualitative study in two districts of Bangladesh, interviews were conducted with 30 bat hunters who hunted bats primarily for consumption, to understand the process and reasons for hunting bats and their perception about bats and bat-borne disease. It was reported that bat meat is prepared for personal consumption and to be sold in neighboring communities. Live bats are also sold to traditional healers. Many informants (53%) reported being bitten by bats. Some informants reported hearing about a disease from bat-contaminated date palm sap, and the NiV was mentioned, but they did not believe that bats could spread a disease to humans because of the absence of disease in their community that have been hunting and consuming bats for generations. However, the close bat–human interaction reported in this study highlights a risk of pathogen spillover [140].

Also, the trade of flying bat meat in markets in Southeast Asia may represent a source of potential infection. For instance, north and southeast areas of the Sulawesi Island in Indonesia are dynamic sites for the wildlife trade of hunted species in wild meat markets, including large amounts of flying foxes that roost are under hunting pressure. However, the current rates of flying fox harvests are significantly unstainable and the impact of this trade on local ecosystems remains mostly unknown [141].

## 7. Biological Cycle/Pathogenesis

Hepanoviruses are the only paramyxoviruses with a zoonotic potential, have a broad host spectrum and are responsible for with high mortality recorded in infected subjects. The virus enters the host via the oral-nasal route and causes the infection of the target cells. The target cells, in which the NiV replicates, were initially unknown, but high concentrations of the NiV antigen were found in the oro-pharyngeal lymphoid tissue (Waldeyer’s ring) and in the respiratory system, identifying them as permissive biological matrixes and primary sites of viral replication [142]. After several studies, viral antigens were found in bronchi and alveoli of experimentally-infected animals which confirmed that the primary target cells are bronchial epithelial cells and type II pneumocytes [143].

Unlike other paramyxoviruses, the NiV is not strictly lymphotropic and tends to infect mainly dendritic cells [144].

The entry of paramyxoviruses into the cell occurs through absorption (mediated by the glycoproteins G, H or HN) and fusion due to the glycoprotein F [37]. Following fusion, viral genes are expressed in the cytoplasm due to the action of the glycoprotein L, the catalytic subunit of the RNA-dependent RNA polymerase (RdRp) together with the nucleocapsid protein N and phosphoprotein P. The assembly and budding take place through the matrix M protein which exploits the inner layer of the cell membrane and recruits the ribonucleoprotein complex and transmembrane glycoproteins for the new virions [38]. The NiV RNA genome is encapsulated in the N nucleoprotein thus forming a long helical assembly [23]. This structure not only safeguards the genome from degradation but also serves as a template for the transcription of the mRNA and the replication of the nascent viral RNA via RdRp [7]. During these stages of replication, the mRNA transcription predominates, and it is transcribed into viral proteins via the host cellular apparatus. Once the required amount of N proteins has been produced, replication of the viral genome begins [145]. The newly synthesized viral RNA is inserted into the capsid via the co-transcription of the new N protein, thus guiding the formation of a further helical nucleocapsid assembly and making the N protein one of the most abundant viral proteins produced throughout infection. During virion formation, the nucleocapsid and the RdRp complex are transported to the plasma membrane for assembly and budding in a process that is primarily driven and coordinated by the interaction of the viral M protein with the assembled nucleocapsid glycoproteins [46,47,142].

During the fusion phase upon receptor binding, the G protein undergoes conformational changes and dissociates from the F protein, which subsequently undergoes a series of conformational changes resulting in the fusion of the viral and host membranes [146,147]. Similar to many paramyxovirus fusion proteins, the NiV F is synthesized as an inactive precursor F0, which is proteolytically cleaved by a host cell protease, endosomal cathepsin L, into the fusion-active F1 and F2 subunits. For the NiV F, this occurs following initial expression at the cell surface and subsequent internalization. The polypeptides are then transported back to the cell surface to be incorporated into budding virions, or to facilitate the cell-to-cell spread of the virus, forming multinucleated syncytia [43,146] which is another characteristic feature of many paramyxovirus infections.

The dissemination of the virus in the host’s organism is facilitated by the binding of the NiV to heparan-sulfates on the cell membranes of circulating leukocytes, and this occurs without directly infecting the cells [148]. Using leukocytes as carriers, the viral particles reach the endothelial cells [144].

After an initial viremia, the virus spreads into the body and replicates in the endothelial cells which represent the secondary site of replication in addition to bronchial epithelial cells and type II pneumocytes. This fusion is pH-independent and mediated by the absorption glycoproteins. The distribution of these receptors explains the clinical-pathological characteristics observed during the clinical signs of the disease. 

The central nervous system is reached primarily through the lympho-hematogenous pathway following endothelial damage at the blood-brain barrier, while a similar mechanism to pulmonary endothelial damage allows the virus to reach the deepest part of the respiratory system [71]. There is also evidence of direct nerve transmission via the olfactory nerve as seen in experimental swine models [149]. The respiratory infection generated by the NiV is induced by the production of inflammatory cytokines that recruit other elements of the immune system, generating a condition similar to acute respiratory distress syndrome (ARDS). The key mediators are interleukin (IL)-6, IL-8, IL-1α, the monocyte chemoattractant protein 1 (MCP-1), granulocyte colony stimulating factor (G-CSF) and granulocyte-macrophage colony stimulating factor (GM-CSF). These mediators are not always expressed in the trachea and bronchi, and this makes the low presence of inflammation in cases of respiratory disease plausible.

The NiV also binds CD3+ leukocytes and in a swine study it was shown to infect monocytes, CD6+CD8+ T cells and natural killer (NK) cells [97].

**Figure 4:**
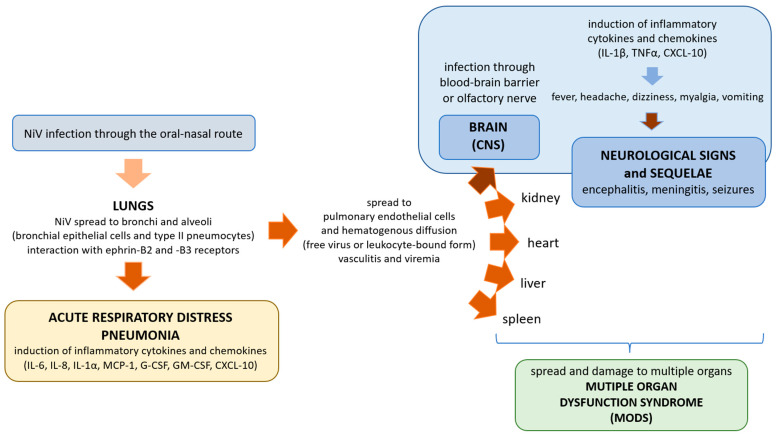
Schematic representation of the NiV pathogenesis. The virus enters the host through the oral-nasal route and spreads into the upper and lower respiratory system, including bronchi, bronchioles and alveoli where bronchial epithelial cells and type II pneumocytes are the major susceptible cells to infection. The first phase of infection is characterized by pneumonia and acute respiratory distress triggered by the release of inflammatory cytokines and chemokines in the lung. Pulmonary endothelial cells are secondary target cells which allow the virus to enter the peripheral circulation and spread into the body as free virus or carried by leukocytes (especially lymphocytes and monocytes), also causing a severe vasculitis and inducing viremia. The virus can reach different organs and induce multiple organ dysfunction syndrome (MODS). The spread to the brain and central nervous system (CNS) induces the release of inflammatory mediators responsible for systemic clinical signs and eventually leading to severe forms of neurological diseases, which mostly lead the host to death or severe impairment. NiV infection can lead to long-term neurological sequelae, late-onset encephalitis or relapse encephalitis after recovering from a symptomatic initial infection. IL: interleukin; MCP: monocyte chemoattractant protein; G-CSF: granulocyte colony stimulating factor; GM-CSF: granulocyte-macrophage colony stimulating factor; CXCL: C-X-C motif chemokine ligand; TNF-α: tumor necrosis factor alpha.

NiV-infected CD6+ lymphocytes interact with endothelial cells through the activated leukocyte cell adhesion molecule ALCAM (CD166) in the microcirculation constituting the blood-air and the blood-brain barrier, but it is not clear how this mechanism allows the NiV to enter the central nervous system. Also, inflammatory cytokines such as TNF-α and IL-1β are involved, likely modifying the permeability of the blood-brain barrier. These cytokines then act at the neuronal and microglia level (Figure 4) [97].

The high lethality of the NiV is attributed to its ability to evade the non-specific immune response. In fact, the products of the P gene were shown to inhibit the activity of interferons [150]. Another study demonstrated the direct inhibition of interferon production [151].

The NiV is transmitted through the droplets of susceptible hosts other than bats [82] and represents the main route of inter-human and inter-animal contagion.

RNA binding by the N protein to form the nucleocapsid is essential in viral assembly and understanding this process would inform the development of antivirals. The N protein is also highly immunogenic, partly due to its high abundance during infection, making it an important tool for the serological monitoring required for diagnostic and epidemiological studies of new and historic outbreaks. Structures of nucleocapsid-like assemblies of several paramyxoviruses, assembled as helical [152], ring [153] or clam-shaped [154] complexes, have been already reported. However, the nucleocapsid of the NiV shares only 32% sequence identity with the nucleocapsid of the measles virus, the closest homologue with an available structure. No structural information is available for the oligomeric assembly of the NiV nucleocapsid, despite the ability of the recombinant protein expressed in bacteria [155], yeast [156] and insect cells [157] to form nucleocapsid-like helical structures containing cellular RNA. New data permit the analysis of similarities and differences with other members of the Paramyxoviridae family as well as more distantly related members from the Mononegavirales order of ssRNA viruses.

## 8. Inflammatory and Immune Response to NiV Infection

In the early stages of the disease in humans, the NiV can be detected in the epithelial cells of bronchi [158]. Viral antigens can be detected in bronchi and alveoli in experimental animal models; the main target cell types seem to be the bronchial epithelial cells and type II pneumocytes [143]. Significant mediators produced in this phase are IL-1α, IL-6, IL-8, G-CSF, GM-CSF, CXCL10 [also known as IFN-γ-induced protein 10 (IP-10)], and MCP-1 [159].

From the respiratory epithelium, the virus spreads to the pulmonary endothelium and subsequently there is a hematogenous diffusion of the pathogen, either freely or bound to white blood cells. In addition to the lungs, the NiV also targets the spleen, kidneys and brain, inducing the multiple organ dysfunction syndrome (MODS) [143,159]. Two routes are used to enter the central nervous system: the hematogenous route (through the choroid plexus or the vessels of the brain) and/or the antegrade route, through the olfactory nerve [149]. In the latter case, infection extends throughout the cribrosa lamina of the ethmoid into the olfactory bulb and finally spreads to the ventral cortex, invading the remaining part of the central nervous system (CNS) [149,159,160]. The entry of the virus into the CNS after infection involves the destruction of the blood-brain barrier and the expression of IL-1β and TNFα, with the consequent onset of neurological signs (Figure 4) [161]. At the cytopathological level in humans, the presence of inclusion bodies and plaques of necrosis is described both at the level of the white matter and gray matter [159].

From an immunological point of view, henipaviruses encode several proteins that block the non-specific immune response and can also act as virulence factors. The NiV P gene is transcriptionally modified to produce not only the phosphoprotein (P) but also the two alternative V and W proteins, while an alternative ORF expresses the C protein [162]. Specifically, phosphoprotein P, proteins V, W and C have the ability to counteract the α/β interferon response. The available data specifically indicate that W and V inhibit both IFN-α/β and IFN-γ responses to various stimuli while P, V and W also block the ability of IFN-β to induce an antiviral state in the cells. Protein V interacts with cytoplasmic RNA-helicases to prevent the activation of IFN-β promoters [163] while protein W inhibits the activation of IFN-β promoters by interfering with the transcription factor IRF3 in the nucleus [164]. None of these factors directly inhibit IFN-β production. Protein C also inhibits the antiviral effects of IFN-α/β via an unknown mechanism [165]. The antagonistic functions of the proteins P, V, W and C were tested in vivo using pathogenesis models in hamsters. These animals after inoculation with the recombinant virus lost the ability to produce both protein C and V, without showing disease and with an almost undetectable viral genome, indicating that the antagonistic properties of these proteins are critical in the pathogenesis of the NiV disease [166].

The immune response of the reservoir host and of the accidental hosts is not yet fully known. Presumably bats generate a robust response to infection which controls the replication of the virus and thus overcomes the disease. The NiV is likely to be able to modulate the immune response to allow for a low level of viral replication and potential persistence by evading the innate immune response as demonstrated in bat cell cultures [151]. This evasion probably gives the virus time to establish an active infection and at the same time modulates the adaptive immune response to allow for low levels of viral replication. It is possible that the virus is extensively cleared by immune mechanisms but persists in minimal amounts in unknown sites for a period of time. This is supported by the difficulty of isolating the virus (or detecting viral proteins with immunohistochemical techniques) from capture or experimentally infected *Pteropus* [167]. In fact, NiV-infected bats seroconvert and generate measurable titers of neutralizing antibodies indicative of a transient infection, whereas those experimentally-infected intermittently eliminate viral agents, demonstrating active replication in these types of hosts [102,167]. The presence of neutralizing antibodies (IgG) suggests that both humoral and CD4+ cellular immune responses are activated during infection [145]. 

As for non-specific immunity, viral RNA released into the cell is recognized by intracytoplasmic RNA-helicases which activate the type I IFN response [168]. Upon recognition, there is a balance between the ability of the cell to activate the innate immune response and the ability of the virus to antagonize such a response. In vitro endothelial cells (important target in vivo) infected with the NiV are able to produce IFN-β and other cytokines and chemokines such as IL-6 and IP-10. IP-10 is a chemotactic factor that activates T lymphocytes, while IL-6 is a cytokine that stimulates the production of acute phase proteins (APPs) [169,170,171]. These cytokines are able to recruit T cells, as demonstrated using in vitro migration tests [172] (Table 2). This probably contributes to the extensive histopathologically reported vasculitis. Expression of innate immunity-related genes is documented as a response to the NiV in experimental models of infection that include the production of IP-10 and IL-6 [161,173].

Further evidence that the nonspecific immune system affects the pathogenesis is obtained from the observation that the synthetic RNA (poly I:C), which significantly activates IFN production, is effective in limiting disease and experimentally increasing the survival rate in NiV-infected hamsters [174].

As for the specific immunity, it is not yet clear whether the adaptive immune response is simply inefficient or whether it somehow triggers the immunopathogenic mechanisms responsible for the clinical-pathological manifestations of the disease. The serum of infected patients contains measurable IgM titers already during the first 4 days after exposure and the subsequent presence of IgG indicates that both B cells and CD4+ T cells are elicited following the presence of the virus [175]. Presumably the disease is, at least in part, the result of a deficient immune response, although the infiltration of immune cells seen in some patients who died from the disease indicates that the recruitment of immune cells, leading to acute vasculitis, can favor the development of the disease [176,177]. The lack of animal models does not allow us to fully elucidate this aspect of the pathogen interaction with the immune system and the only ones that partially mimic the human immune system are Syrian hamsters, ferrets and cats [178,179,180]. A NiV disease model using non-human primates was described and seems to properly mimic the disease in humans but is underutilized for obvious reasons [181]. Immunocompromised murine models that can develop the disease could be particularly helpful (IFNAR knockout or STAT1 knockout mice), but their use is still limited [182,183].

A useful contribution to the study of specific immunity during NiV infection is given by studies for the development of vaccines against the disease. The aim of the numerous vaccines studied, coding for the surface glycoproteins G or F (or both), is to elicit the production of neutralizing antibodies. The efficacy of these vaccines suggests that the effective immune response against NiV greatly benefits from the presence of neutralizing antibodies. Furthermore, prophylactic or post-contagion administration of hyperimmune sera or monoclonal antibodies was shown to be useful in experimental disease models in both hamsters [184] and ferrets [180]. The same antibodies also protect Hendra-infected non-human primates when administered post-infection [185]. While neutralizing antibodies are highly protective when administered before or shortly after infection, the role that specific immunity plays in both vaccination-induced protection and natural immunity is still unclear.

However, it was demonstrated that survivors of NiV infection in the 2018 NiV outbreak in India had detectable levels of IgM and IgG antibodies, and increased B lymphocytes in the blood, likely responsible for protection during acute and delayed infection phases [186]. Conversely, bats have naïve immunoglobulins with high specificity, therefore they could control NiV replication by B cell expansion and lower amounts of antibodies, which could sustain viral persistence and spread for long periods [50].

Currently, no vaccination strategy has been used to address this problem, partly due to the lack of syngenic cell lines to measure cytotoxic T lymphocyte (CTL) activity, or antibodies to measure CD8+ and CD4+ cell responses by flow cytometry, or to measure cytokines in these animal models. 

In humans, scarce data are available due to the limitation of samples due to the high virulence and death of infected people. However, the analysis of the above-mentioned Indian survivors demonstrated that T cells levels did not change in the blood but increased frequencies of activated CD8+ cells expressing granzyme B, Ki67 and PD-1 were detected. This highlights the importance of the cytotoxic/cytolytic cellular response in achieving viral clearance [186].

To study the contribution of the cellular response, one approach could be to develop and measure the efficacy of vaccines that include, or encode, both non-structural proteins of the NiV and parts of structural proteins that do not contain epitopes that induce neutralizing antibodies. Interestingly, one way the immune response can exacerbate the disease is by spreading the virus. Indeed, viable NiV was shown to be carried by lymphocytes and monocytes without effectively infecting or possibly even entering these cells, only to be released at sites distant from the initial infection site [144]. This may be one way by which the immune system actively participates in the spread of the virus and may explain the widespread viral distribution observed despite little or no detectable viremia in disease models. Furthermore, porcine monocytes, NK cells and CD8+ T cells were reported to support NiV replication, thus facilitating virus spread [187].

However, African green monkeys and pigs surviving NiV experimental infection showed increased CD4+ and CD8+ effector memory cells and/or increased activated T helper memory cells. Mice immunized with NiV F and G proteins mounted antigen-specific CD4+ and CD8+ cell responses, suggesting the importance of these cellular subsets in eliciting a protective immune response (Table 3) [50].

## 9. Clinical Signs

### 9.1. Chiroptera

Despite the high seroprevalence as reported in the OIE Terrestrial Manual 2022 [188], where several studies are cited, including those by Rahman et al. [189,190] which report a seroprevalence of up to 75% in bats of the genus *Pteropus*, the disease evolves in an asymptomatic manner. To confirm the presence of the pathogen in reservoir bats, studies carried out in Thailand detected the virus in the biological matrixes of completely asymptomatic subjects, such as in the saliva, blood and urine, using RT-PCR techniques [191,192].

### 9.2. Pigs

In pigs, the disease is characterized by respiratory and neurological clinical signs of varying severity depending on the age of the affected subject. In suckling pigs (less than 4 weeks of age), mortality can reach 40% and dyspnea and muscle tremors are the most evident symptoms. In animals aged one-to-six months, the disease occurs with fever (>39 °C), respiratory signs ranging from increased respiratory effort to dry non-productive cough, “open mouth” breathing and epistaxis [188]. In some cases, respiratory signs are associated with one or more of the following neurological signs: tremors, muscle fasciculations, tetanic spasms, motor incoordination, posterior weakness and paraplegia. Subjects of this age have high morbidity and low mortality (<5%) [2,193]. Some animals older than six months of age die rapidly within 24 h without any clinical signs. In adults, respiratory disease such as that of piglets is mainly described but definitely less severe (increased respiratory effort, nasal discharge, sialorrhea) and neurological signs such as head in the corner, biting the bar, tonic-clonic muscle spasms and seizures can occur. Early miscarriages are also reported [2,193]. In experimentally-infected pigs, after subcutaneous inoculation of the NiV isolated from the CNS of human subjects who died of the disease, the subjects showed symptoms referable to fever, motor incoordination, nasal discharge and cough. Subjects inoculated orally with the same dose of the NiV showed no clinical signs although viral particles were recovered from the tonsillar epithelium and upper respiratory tract [178]. In another study, piglets orally-infected with human NiV isolates showed transient increases of body temperature between 4 and 12 days after injection, associated with mild respiratory and neurological symptoms in some subjects [194].

The proposed names for the NiV disease in pigs are swine respiratory and neurological syndrome (also known as swine respiratory syndrome and encephalitis) or, as it is called in Malaysia, barking pig syndrome (BPS) [2].

### 9.3. Dogs and Cats

In dogs, the symptoms are characterized by respiratory distress (cough, dyspnea) and renal disfunction (polyuria/polydipsia, oliguria) as well as by fever, vomiting, depression and dehydration [5].

Cats can be highly susceptible to NiV infection and can develop fever, depression, respiratory distress (increased respiratory rate, dyspnea with prolonged expiratory phase and open-mouth breathing), vomiting, dehydration and jaundice [178].

### 9.4. Humans

The incubation period in humans ranges from 4 days to 2 months, with most subjects (>90%) developing symptoms within 2 weeks after exposure to NiV [89]. Patients can have fever, headache, dizziness and vomiting which then progresses to severe encephalitis. Many patients show sensory blunting and severe signs of medulla oblongata dysfunction in which symptoms are attributable to abnormal pupillary reflexes, vasomotor changes, seizures and myoclonus prevail [89]. Neurological involvement is varied and multifocal, and includes meningitis, diffuse encephalitis and focal involvement of the medulla oblongata. Cerebellar signs are relatively common [86]. A unique feature of NiV infection is the development of relapse and late onset of encephalitis in survivors, even months or years after the acute onset [195]. Other manifestations may be psychiatric, such as depression and personality changes or attention deficit, as well as verbal and/or visual memory deficit [196].

Neurological manifestations are well-documented in severe cases of human outbreaks in Malaysia with development of ARDS (50–60%) especially in the advanced stages of the disease [87].

Although asymptomatic infections are well-documented, data are lacking on what percentage of patients remain asymptomatic. The incidence of subclinical infections varies from 1% to 45% in various studies [99]. Vomiting, dysphagia and myalgia are also described [6].

Having a high index of suspicion and awareness about uncommon manifestations in high-risk areas is important to diagnose NiV infection [197].

## 10. Differential Diagnosis

### 10.1. Swine

There are no pathognomonic clinical signs of NiV infection in pigs, making the clinical diagnosis difficult to interpret, particularly considering the numerous differential diagnoses that the clinician must consider, such as: classical swine fever (CSF virus), African swine fever (ASF virus), porcine reproductive and respiratory syndrome (PRRS virus), Aujeszky’s disease (AD virus), enzootic pneumonia (*Mycoplasma hyopneumoniae*), pleuropneumonia (*Actinobacillus pleuropneumonia*) and pasteurellosis [198].

### 10.2. Humans

In humans, the main differential diagnoses to be considered are malaria, Japanese river typhus, leptospirosis, dengue, herpetic meningoencephalitis, bacterial meningitis, Japanese encephalitis, measles and rabies [87].

## 11. Clinical Pathology/Imaging

The early diagnosis of NiV encephalitis, in both humans and animals, is complex and represents a crucial step in the management of infections caused by the pathogen. Suspicious diagnoses in humans in the hospital setting can be issued, such as leukopenia, thrombocytopenia, increased liver enzymes and hyponatremia, following laboratory findings in addition to clinical data [87].

In humans, the biological matrixes to be collected for ancillary laboratory investigations, in vivo, are nasal swabs, throat swabs, urine, blood and cerebrospinal fluid (CSF), while during *post mortem* (humans and animals) they are collected from the lung, spleen and kidney [199]. Samples for serological testing should be collected in the late stage of the disease, approximately 10–14 days after the onset of symptoms [3].

Diagnosis should ideally be made in a biosafety level 4 (BSL-4) laboratory, although a BSL-3 may be sufficient to primarily isolate the virus from suspicious clinical material. In fact, following the confirmation of the presence of viruses in the infected cell lines, there will be an immediate transfer to a higher biosecurity laboratory [199].

In the first Malaysian outbreak, CSF was abnormal in 75% of patients, with an increased concentration of protein or an increase in white blood cell count. CSF glucose concentrations were within normal limits [89,200]. However, these are non-specific findings that can be found in encephalitis of different etiology.

A valuable aid in the diagnosis of suspicion is magnetic resonance imaging (MRI) in patients with acute encephalitis [201]. Usually with this type of patient there are multiple, circular, small hyperintense lesions, more accurately detected by fluid attenuated inversion recovery (FLAIR), located mainly in the deep subcortical white matter and occasionally in the cortex. These lesions, which measure from about 2 to 7 mm in diameter, correspond to the micro infarcts detected in the autopsy. Similar changes are also observed in 16% of asymptomatic patients [202].

## 12. Diagnosis

The different methods for the etiological diagnosis of NiV infection include serological, molecular, virological and immunohistochemical (IHC) methods [93].

The main different methods to determine virus infection and immunity are:

- Enzyme-linked immunosorbent assay (ELISA): it is used to detect the NiV antigen and also to evaluate the antibody response. It is a simple and inexpensive method for screening suspicious samples [5]. Several techniques of this serological test are used: ELISA-capture uses monoclonal antibodies for the detection of the NiV and to differentiate it from the forms of HeV [203] or even from the one that uses the recombinant N protein of the NiV [204]. Indirect ELISAs for IgG and IgM have also been developed to test both porcine and human sera [2] and are also described to detect seroconversion in bats [205,206]. Another variant of the technique is a sandwich ELISA, which uses rabbit polyclonal antibodies against the NiV G protein [207]. Among the tests used for screening in suids, one based on the use of a recombinant protein N was developed in India (High Security Animal Disease Laboratory [HSADL], Bhopal, India) [5].
- Virus neutralization test (VNT): this test was developed shortly after the Malaysian outbreak and was considered the reference serological test. The conventional NiV VN test commonly uses Vero cells where the prevention of the cytopathic effect via the serum to be tested is considered as a positive neutralization. Furthermore, plate VN tests were developed [208].

The neutralization test was also developed by using a pseudo-type vesicular stomatitis virus containing NiV envelope proteins in order to be neutralized using a serum containing specific antibodies [209]

- Molecular biology methods: the most sensitive and specific system is PCR. The viral N, M and P sequences are often the targets of RT-PCR and nested-PCR [6]. Furthermore, NiV-targeted PCR has important applications in phylogenetic studies [199,206]. RT-PCR (and its variants) represent the gold standard for the detection of the NiV from various biological samples. RT-PCR for the NiV was developed in 2004 and is based on the N gene sequence. It had high specificity as it was able to detect NiV RNA in blood samples from infected hamsters, where the HeV could not be detected [210]. A commercial kit, developed by Zoologix Inc. (Chatsworth, CA, USA), is able to detect the NiV in whole blood, plasma, CSF and infected tissues/secretions from different animal species [211]. These tests are vital for viral surveillance; for example, the nested RT-PCR duplex, used in Thailand on specimens of *P. lyei* and which distinguished the NiV variants circulating in bat populations [23,184,192]. Other reported RT-PCR techniques include the SYBR Green quantitative real-time PCR (qRT-PCR) using N-specific primers [212], and a novel one-step qRT-PCR that targets the inter-genic region between the F and G gene sequences for the quantitative detection of replicative NiV RNA, discriminating from the mRNA, which may be more accurate than the conventional qRT-PCR [213].
- Viral isolation: it is very useful in early cases and in new outbreaks where the NiV is suspected. Samples are brain, lung, kidney and/or spleen. Vero cells are a suitable substrate for NiV growth, and the cytopathic effect is usually observed after 3 days of culture in the form of characteristic syncytia and plaques in the cell monolayer [6]. The next step in virus identification includes immunostaining, seroneutralization (SN) and PCR of the culture supernatant. Electron microscopy and immune electro-microscopy are useful tools for identifying the structure of the NiV and detecting virus-antibody interactions, respectively [199,205].
- Immunohistochemistry (IHC): anti-NiV antibodies have been used to stain formalin-fixed tissues of the CNS, lung, spleen, lymph nodes, kidney and heart for detecting viral antigens. In tissue sections, it is possible to identify NiV-associated lesions such as flogosis, necrosis and vasculitis [206].

Diagnostic methods for henipaviruses and the anti-viral immune response applied to specific conditions related to humans and animals (i.e., population free of disease, movement of uninfected animals, eradication policies, confirmation of clinical cases, prevalence of infection-surveillance, and immune status of the animal/human population in case of vaccination), as well as the purpose of the application of the methods, are detailed and updated in the OIE Terrestrial Manual 2022 [188].

## 13. Pathology

### 13.1. NiV in Natural Host—Pteropus Bats

Natural NiV infection induces seroconversion in *Pteropus* bats but does not induce severe lesions because they are the natural host, thus a NiV reservoir in which the virus is not subjected to strong evolutionary shedding pressure as it can be easily spread among bats thanks to bat ecology itself. During NiV infection under experimental conditions, bats develop a subclinical infection characterized mainly by focal vasculitis, mild chronic interstitial nephritis and focal hepatic cellular infiltration [167].

### 13.2. Pigs

Upon natural NiV infection, the most relevant lesions are detectable in pulmonary parenchyma which appears diffusely firm, with large lobar consolidation and reddish discoloration. Lung lobuli are distended due to the pressure induced by inflammatory fluids collection on the septal wall. The trachea and bronchi lumina can be partially occluded by foamy inflammatory fluids. Regional lymph nodes of respiratory stations appear megalic and haemorrhagic [214]. Diffuse haemorrhages, sometimes coalescent, are also detected in the pulmonary and renal parenchyma. Histopathological examination shows a polyvisceral vasculitis, often thrombosis associated. Foci of necrotic epithelial alveolar cells, as an outcome of viral lytic replication, are observed. Lung pneumonia is a giant-cell pneumonia with symplastic multinucleated cells recognizable in the respiratory epithelium as the cytopathic effect in NiV-permissive cells [23]. Symplasmic cells, syncytia cells, or polykariocytes are different terms for the same pathological phenomenon of NiV-permissive epithelial and/or endothelial cells subjected to viral infection. Syncytia formation is due to the interaction of NiV virions with permissive cells (i.e., endothelial and pulmonary). Adjacent cells fuse in one giant multinucleated cell called a syncytia cell, or symplastic cell. Syncytial endothelial cells and syncytial smooth muscle cells were observed in veins and arteries that supply the CNS, and the respiratory and lymphoid systems [149,176,178]. Syncytia are also present in the lymphatic vessels and lymphangitis is often associated with lymphoid tissue necrosis and/or lymphocyte depletion [214,215]. In experimentally NiV-infected pigs, lymphocyte depletion involves the CD4+CD8+ T lymphocyte subset and it concerns pigs that have died [187].

Perivascular mononuclear infiltration (“perivascular cuffing”) is the most important histopathological feature detected in pig’s CNS affected by nervous disfunction [214]. 

In an experimental pig model in which pigs were sensitive to the Bangladesh NiV strain, gross pathology and histopathology features were very similar to those observed upon natural infection. In addition, renal tubular degeneration was observed after the first week of onset of NiV-related classical respiratory signs. A study performed on a biological matrix collected from experimentally-infected pigs showed NiV-positivity in different tissues (upper respiratory tract, tonsil, pulmonary parenchyma, lymphoid system, endothelial cells, olfactory bulb, brain, kidney) and biological fluids (blood, urine). NiV immunolabeling was observed in epithelial cells of the lower respiratory tract, endothelial syncytial cells, lymphoid cells and renal glomeruli and renal interstitium [178].

Ultramicroscopy (TEM) studies of the respiratory tract showed viral particles or aggregates of nucleocapsid in epithelial bronchial cells and syncytia cells [178].

### 13.3. Dogs and Cats

In dogs, gross pathology is characterized by lung edema and multisystemic vasculitis. Histopathology highlighted the presence of syncytia, necrosis, glomerulonephritis and meningitis [176].

Gross pathology is characterized by interstitial pneumonia and diffuse vasculopathy of the lung parenchyma, gastro-intestinal tract and lymphoid system. Upon histopathology examination, syncytia of endothelial cells were observed [178,216].

Cats can develop ganglioneuritis during the acute clinical disease, upon experimental infection with the NiV [178]. In pregnant NiV-infected cats, the placenta is NiV-permissive and can be infected as well as the fetal tissues. The NiV can be recovered from the placenta and uterine fluid [217].

### 13.4. Horses

In horses, lung lesions are marked by haemodynamic pulmonary oedema secondary to vasculitis. Lungs are characterized by a reddish discoloration of the surface associated to interstitial and septal oedema or gelatinous bands with separation of septa [72,176,216].

### 13.5. NiV in Animal Models

Ferrets, immunodeficient mice, hamsters and guinea pigs are NiV-permissive animals and gross pathology and histopathology is similar in terms of target tissues and severity in naturally NiV-susceptible hosts [218]. In immunocompetent mice, almost absent and self-limiting lesions in the respiratory tract were recorded [182]. Rats, rabbits and chickens are NiV resistant animals. Chicken embryo is NiV-permissive when inoculated in the allantoid or yolk sac, and the most important lesions are related to blood vessels (multi-organ congestion and hemorrhages). Histopathology points out the presence of syncytial cells and necrosis [219].

### 13.6. NiV in Non-Human Primate Models: African Green Monkeys (AGM) and Squirrel Monkeys (SM)

Multi-organ vasculitis, hemorrhages and oedema are the most consistent gross lesions in African green monkeys. Thrombocytopenia and meningitis are also pathological features detected by using laboratory ancillary exams [181]. The Bangladesh NiV isolate is more histolesive in the lung and spleen than the Malaysia NiV isolate upon experimental infection [220]. In squirrel monkeys, diffuse mild vasculitis and pneumonia are the most relevant lesions observed [221].

## 14. Prevention

### 14.1. Biosecurity

Since the NiV is a virus for which there is no specific standardized therapy and vaccines produced on a large scale, biosecurity, intended as a reduction of the risk of transmission to humans and animals, represents a cornerstone in the prevention of any disease outbreaks.

For operators in both the agricultural and livestock fields, good practices in pig farming and the protection of crops from contamination by bats play a fundamental role in terms of prevention. Another very important point is the hygiene of the staff intended, above all, the simple but effective hand method of washing. If an outbreak is suspected, it is good practice for personnel to wear personal protective equipment (PPE) such as masks, gloves, protective goggles, gowns and boots which must be thoroughly washed and disinfected after use [222,223].

### 14.2. Vaccines Candidates and Available Vaccines

So far, no vaccines against NiV infection in humans have been produced and are available as the difficulty of dealing with a highly contagious and virulent virus is one of the concerns together with the opportunity of collecting human samples during the outbreaks which occur in the countries where NiV virus spreads.

The effectiveness of a vaccine is often not only dependent on the induction of high levels of neutralizing antibodies but also on a robust cellular response, especially in terms of memory B and T cells able to efficiently counteract acute infection and re-infections during the lifespan.

Regarding henipaviruses, administration of purified antibodies derived from the serum of convalescent individuals have been tested against infection, as passive immunization can be efficacious for the treatment of infected subjects. Only two studies involved reagents that may be utilized in humans in the future as immunotherapy: a cross-reactive human monoclonal antibody (m102.4) was tested in vivo in NiV-infected ferrets and AGM, showing a neutralizing activity against the NiV G protein (main antigenic determinant) and neutralizing the interaction with its receptor [3]. This antibody was tested in a phase-1 human study in which it was administered as single and multiple doses and proved to be safe and well-tolerated [224]. Another cross-reactive humanized monoclonal antibody (h5B3.1) was developed and showed a degree of protection in ferrets. In this case, the antibody neutralized the action of the NiV F protein (responsible for the fusion with the target cell membrane). 

Other vaccine strategies are under study, such as the administration of subunit vaccines and live viral vector-based vaccines. The use of live-attenuated virus vaccines, despite its potential efficacy in stimulating higher levels of immunity, is difficult to achieve because of the high virulence and mortality related to the NiV. Therefore, a subunit vaccine based on the soluble G protein of the NiV and HeV has been studied in animal models and provided promising results in terms of protection and cross-protection [10,11].

A vaccine based on a recombinant and soluble form of the HeV protein G (Equivac^®^ HeV; Zoetis, NJ, USA) was the first available on the market and is currently licensed in Australia to protect horses against HeV and reduce the zoonotic risk [25]. This vaccine also immunizes ferrets and AGM after experimental NiV infection in the same way as that against HeV [225,226]. This product is not similarly effective in pigs experimentally-infected with the NiV [227]. Nowadays, the HeV subunit vaccine is under clinical development as an emergency vaccine for NiV infections [228].

Experimental viral-vectored vaccine candidates for pigs include vesicular stomatitis virus (VSV), canarypox virus (CNPV, ALVAC strain), adeno-associated virus (AAV), measles virus (MV), Newcastle disease virus (NDV), Venezuelan equine encephalitis virus (VEEV) and bovine herpesvirus 4 (BoHV-4) [7,8,9].

The CNPV ALVAC strain expressing NiV G or F protein seems to be protective in pigs at 2 weeks after the second administration [12]. ALVAC-G induces a higher production of neutralizing antibodies than ALVAC-F, and all vaccinated animals in the aforementioned study were protected against experimentally inoculated virulent NiV strains.

Despite some encouraging results, no viral vector vaccine is currently in the process of being produced to contest the disease in pigs [198]. BoHV-4-based vector vaccines expressing either the G or F NiV protein proved to induce virus-neutralizing antibodies and CD4+ and CD8+ T cell responses comparable with those elicited by the ALVAC vaccine [9]. A recombinant MV-vectored vaccine expressing the NiV G protein (rMV-NiV-G) showed promising protection in AGM and may be a good candidate for use in humans [13].

Recent research on a tetrameric NiV G protein-based vaccine in rhesus macaques revealed its pivotal role in stimulating the immune response in terms of neutralizing antibodies and may be the cornerstone for future vaccines with enhanced immunogenicity as with SARS-CoV-2 [229].

The same as for SARS-CoV-2, new technologies based on artificial intelligence (AI) analysis helped researchers investigate and better understand interconnected aspects of NiV infection such as virulence, transmission modes and genotype-related molecular features of the viral proteins N and M. By using the neural network PONDR^®^ VLXT as an AI tool [230] for shell protein disorder prediction and evaluation, it was demonstrated that different percentages of intrinsic disorder (PID) (i.e., no unique 3D structure in a protein or in a protein region which is reflected in new functions) in the N protein, and therefore genetic diversity, is correlated to a different case/fatality ratio (CFR) which is a measure of virulence. Different shell disorder in the viruses responsible for the outbreaks in different countries suggests a link with the modes of transmission and virulence/infectivity. These analyses can help design and develop new potential attenuated vaccines and prevent future outbreaks based on the knowledge of the predicted transmission mode [231,232].

## 15. Legislation

### 15.1. Legislation in Europe

In Europe, the relative legislation refers to the generic management of communicable diseases, and specifically to Art. 6 of the Regulation 2016/429 for emerging diseases [20]. If NiV infection should occur in the European Union (EU) for the first time, the Commission would be empowered to adopt an urgent implementing regulation. The next steps would be the modification of the regulations 2018/1629 and 2018/1882 to put it in the list of reportable diseases and categorize it [233,234].

In Italy, at the moment, there are no specific regulations, and we refer to the national and community legislation for the generic management of communicable diseases.

### 15.2. Legislation in India and Malesia

Before the recent outbreak which occurred in Kerala (India), in April 2021, the Indian National Centre for Disease Control (NCDC) [22] had established guidelines for the definition of suspected, probable and confirmed cases of NiV infection in order to early detect and control the outbreaks:

- a “suspected case” is defined by a person from a community affected by a NiV outbreak showing fever and an altered mental status or respiratory signs;
- a “probable case” is defined by a person who lived in the same area where a confirmed case was detected during an outbreak and died before diagnosis, or a person who came in contact with a confirmed case in the hospital (nosocomial infection) who died before diagnosis during an outbreak;
- a “confirmed case” is defined by a person who was a “suspected case” whose infection was confirmed by diagnostic tests (PCR for viral RNA or virus isolation from biological fluids) [3].

Following the outbreak in 2021, the Government of India ordered the following public health activities:

- the state government held a meeting of senior health officials to plan and implement response measures;
- a district central committee was formed and a district action plan for the NiV disease was published for all interested parties;
- a central multidisciplinary team from the NCDC was sent to the state of Kerala to provide technical support. Immediate public health measures were applied, including an active search for cases in families, hospitals, villages and areas with similar topography, especially in the Malappuram district, located in the south-east of the Kozhikode district;
- risk communication campaigns were conducted regarding the transmission of the NiV disease and prevention measures;
- national authorities issued an alert to the districts of Mysuru, Mangaluru, Chamarajanagar and Kodagu in the state of Karnataka, which border the state of Kerala.

In Malaysia, as reported by Aziz and Azri in 2011 [21], following the NiV outbreaks in pigs of the late 1990s, a series of provisions aimed at containing the spread of the infection were planned.

Specifically:

- the isolation of potential disease transmitters;
- the blocking of pigs and meat trading (both locally, nationally and internationally);
- restrictions communicated through the media;
- the observation of restrictions strictly controlled by law enforcement and veterinary services;
- the movement of pigs outside the infected areas to state slaughterhouses being allowed as long as there is sanitary authorization from the veterinary services who will escort the animals during the commute, supervising them;
- the establishment of an infected area (2 km) and a protection area (10 km) around the outbreaks. The slaughter of pigs will be carried out with the involvement of the veterinary services, the department of transport, the army and other government agencies and non-governative organizations (NGO);
- the animals will be stunned and killed by bullet and then buried deep in designated areas both on the farm and outside, as appropriate;
- after the killing, cleaning and disinfection operations with chlorine will be carried out on the farm and the sites where the stamping-out is physically performed.

A surveillance program was implemented to establish the status of the pig population on farms in Peninsular Malaysia following the turn-of-the-century outbreaks [21]. 

Following the tests, the animals were killed in a further 50 NiV-positive farms [2]. 

These actions made the peninsular Malaysian pig population free from the NiV in 2001. The subsequent phases of the control and eradication programs included the screening of the pigs sent to slaughterhouses which were traced by means of individual identification which aimed to trace the original holding facility. They were tested, using serological tests and according to established criteria and programs in order to have a clearer picture of the epidemiological situation in the original holding facility. The important purpose of these surveillance programs was to demonstrate that the NiV did not circulate in companies in order to restore confidence in consumers of pork and its products [21]. 

## 16. Conclusions and Future Perspectives

The NiV is considered an emerging pathogen responsible for a zoonosis with epidemiological characteristics relating to high mortality. Bats, as natural reservoirs of the virus, are responsible for the viral spread and, therefore, for the outbreaks of the disease in humans and animals. Due to the worldwide distribution of bats, potential new reports as well as new spillovers are foreseeable in the future.

The NiV may pose a potential risk of spread to humans and animal husbandry in an area of the world where economic migrants from NiV outbreak locations/neighborhoods are in temporary or long-term employment situations, especially when they come in contact with NiV-permissive livestock (e.g., pig farms). Accurate monitoring of migrant entries from areas of NiV outbreaks or borders should be adopted as a health measure in compliance with specific national or international health security regulations and procedures to cope with the spread of the NiV into uncontaminated world areas.

The high mortality rate upon infection and its acute course makes the disease difficult to diagnose, especially in countries with “weak” economies, since they do not have equipped and easily accessible diagnostic facilities. Furthermore, diagnostic kits are still expensive.

A specific treatment and an effective prophylaxis have not been yet fully developed due to the still few targeted studies on humans related, above all, to the pathogenetic and immunological aspects of the disease.

Clear action and surveillance plans are essential, especially in Southeast Asia. In these conditions of high epidemiological risk due to “One Health” problems, the intervention of the WHO with the allocation of economic resources (e.g., equipped laboratories and related consumables for the operations, hospital departments) as well as professionals (e.g., doctors, veterinarians, and laboratory technicians) with NiV-oriented training will be the appropriate and economically sustainable responses to geographically contain the risk and mitigate the impact on worldwide health.

The drafting of new surveillance plans, the organization of training internships, dedicated to doctors and veterinarians, even in the presence of areas at risk of NiV infection, are the concrete answers to the challenges that await us in future years and in which the wildlife expert veterinarians will certainly play a decisive role in the epidemiological scenarios of NiV outbreaks and in those countries that have direct/indirect relationships with those of Southeast Asia at NiV risk.

In addition, particular attention should be given to climate and anthropogenic changes which are known to play important roles in the spread of viral infections to animals and humans, as well as in the maintenance of wildlife and biodiversity, especially when the wildlife habitat is subjected to significant losses. The compromising of wildlife habitats can lead to increased spillover events of zoonotic pathogens such as the NiV, spreading from wildlife to domestic animals and humans, potentially leading to the emergence of new zoonotic outbreaks.

## Figures and Tables

**Table 1:** NiV genotypes and routes of transmission based on the countries where outbreaks occurred [6,72,93].

	Genotype	Initial Source of Infection	Intermediate Host	Source of Infection for Humans	Transmission
Malaysia Singapore	NiV-M	Bat-bitten fruit	Pig	Pig excretions Pig secretions Aerosol Raw meat/products Direct contact Aerosol Fomites	Pig-to-human (main route) Human-to-human
Bangladesh	NiV-B	Bat saliva-contaminated and excreta-contaminated palm sap	–	Raw palm sap Direct contact Aerosol Fomites	Bat-to-human Human-to-human (especially nosocomial)
India	NiV-B	Bat-bitten fruit	–	Fruit Direct contact Aerosol Fomites	Bat-to-human Human-to-human (especially nosocomial)
Philippines	NiV-M	Bat-bitten fruit (most likely)	Horse	Direct contact Horse secretions Raw meat Direct contact Aerosol Fomites	Horse-to-human Human-to-human (also nosocomial)

**Table 2:** Main features of innate immune modulation upon NiV infection.

Inflammation and Innate Immunity	References
- Induction/suppression of type I interferon (IFN-α and IFN-β) production	[50,165,172]
- Induction of IFN-related antiviral genes: RSAD2, CXCL-10, ISG56, OAS1	[50,165]
- Modulation of STAT1 and STAT2 proteins, and ISGF3 transcription factor complex	[50,165,172]
- Modulation of STAT4 and STAT5 proteins, and MDA5 activator	[50,165]
- Induced degradation of TRIM6 protein	[50,165]
- Stimulation of pro-inflammatory cytokines and chemokines (TNF-α, IL-1α, IL-1β, IL-6, IL-8, MIP-1α, MCP-1, G-CSF, GM-CSF, CXCL-10, APP)	[50,159,172]

IFN-α: interferon alpha; IFN-β: interferon beta; RSAD: radical S-adenosyl methionine domain containing; CXCL: C-X-C motif chemokine ligand; ISG: IFN-stimulated gene; OAS: 2′-5′-oligoadenylate synthetase; STAT: signal transducer and activator of transcription; ISGF: IFN-dependent positive-acting transcription factor; MDA: melanoma differentiation-associated; TRIM: tripartite motif-containing; TNF-α: tumor necrosis factor alpha; IL: interleukin; MIP-1α: macrophage inflammatory protein 1 alpha; MCP: monocyte chemoattractant protein; G-CSF: granulocyte colony stimulating factor; GM-CSF: granulocyte-macrophage colony stimulating factor; APP: acute phase proteins.

**Table 3:** Main adaptive immune responses and immune modulation upon NiV infection.

Adaptive Immunity	References
Antibody and B cell responses	
Humans:	
- Induction of NiV-specific IgM and IgG antibodies in serum within 1 week after exposure	[50,175,186]
- Increased counts of B lymphocytes, correlated with NiV-specific IgM and IgG	[50,165,186]
Experimentally-infected pigs:	
- Induction of VN antibodies within 1 week post-infection, increasing over time but slow viral clearance	[50]
Experimentally-infected AGM:	
- Variable development and duration of the B cell response, correlated with disease progression or survival	[50,144]
- Development of IgM and IgG responses, and low levels of VN antibodies in survived animals	[50]
- Increased counts of B lymphocytes, correlated with increased antibody levels	[50]
T cell responses	
Humans:	
- Increased levels of activated CD8+ cells expressing granzyme B, Ki67, and PD-1	[50,165,186]
- Lymphocytes, monocytes, and DC carrying the virus in the host, thus supporting viral spread	[159,187]
Experimentally-infected pigs:	
- Increased activated Th memory lymphocytes (CD4+CD8+CD25+) and CTL (CD4-CD8+CD25+) - Infection and depletion of CD4+ Th lymphocytes and CD8+ CTL	[50,165,187]
Experimentally-infected AGM:	
- Increased CD4+ and CD8+ effector memory cells, correlated with increased Th1 cytokines and chemokines - Increase in NK cell proliferation and functional activity during acute and convalescent phases in survived animals	[50,165]

Ig: immunoglobulins; AGM: African green monkeys; VN: virus neutralizing; PD: programmed death; Th: T helper; DC: dendritic cells; CTL cytotoxic T lymphocytes; NK: natural killer.

## Data Availability

Not applicable.
